# Reliability of classification for post-traumatic ankle osteoarthritis

**DOI:** 10.1007/s00167-015-3871-6

**Published:** 2015-11-26

**Authors:** Femke M. A. P. Claessen, Diederik T. Meijer, Michel P. J. van den Bekerom, Barend D. J. Gevers Deynoot, Wouter H. Mallee, Job N. Doornberg, C. Niek van Dijk

**Affiliations:** Orthopaedic Hand and Upper Extremity Service, Yawkey Centre, Massachusetts General Hospital, Harvard Medical School and University of Amsterdam Medical School, 55 Fruit Street, Boston, MA 02114 USA; Orthotrauma Research Centre Amsterdam, Academic Medical Centre Amsterdam, Amsterdam, The Netherlands; Orthopaedic Surgeon, Shoulder and Elbow Unit, Onze Lieve Vrouwe Gasthuis, Amsterdam, The Netherlands; Department of Orthopaedic Surgery, Academic Medical Centre Amsterdam, Amsterdam, The Netherlands

**Keywords:** Ankle trauma, Post-traumatic ankle fracture osteoarthritis, Classification system, Interobserver study

## Abstract

**Purpose:**

The purpose of this study was to identify the most reliable classification system for clinical outcome studies to categorize post-traumatic—fracture—osteoarthritis.

**Methods:**

A total of 118 orthopaedic surgeons and residents—gathered in the Ankle Platform Study Collaborative Science of Variation Group—evaluated 128 anteroposterior and lateral radiographs of patients after a bi- or trimalleolar ankle fracture on a Web-based platform in order to rate post-traumatic osteoarthritis according to the classification systems coined by (1) van Dijk, (2) Kellgren, and (3) Takakura. Reliability was evaluated with the use of the Siegel and Castellan’s multirater kappa measure. Differences between classification systems were compared using the two-sample *Z*-test.

**Results:**

Interobserver agreement of surgeons who participated in the survey was fair for the van Dijk osteoarthritis scale (*k* = 0.24), and poor for the Takakura (*k* = 0.19) and the Kellgren systems (*k* = 0.18) according to the categorical rating of Landis and Koch. This difference in one categorical rating was found to be significant (*p* < 0.001, CI 0.046–0.053) with the high numbers of observers and cases available.

**Conclusions:**

This study documents fair interobserver agreement for the van Dijk osteoarthritis scale, and poor interobserver agreement for the Takakura and Kellgren osteoarthritis classification systems. Because of the low interobserver agreement for the van Dijk, Kellgren, and Takakura classification systems, those systems cannot be used for clinical decision-making.

**Level of evidence:**

Development of diagnostic criteria on basis of consecutive patients, Level II.

**Electronic supplementary material:**

The online version of this article (doi:10.1007/s00167-015-3871-6) contains supplementary material, which is available to authorized users.

## Introduction

Radiographic evidence of osteoarthritis of the ankle may be associated with unsatisfactory ankle function. Osteoarthritis of the ankle joint varies based on aetiology (i.e. primary, secondary to inflammatory disease, or post-traumatic due to chronic instability, osteochondral defects, or post-fracture osteoarthritis), and radiographic appearance of degenerative changes might similarly differ based on the underlying condition [1, 35]. It might be that radiographic appearances as well as reliability of current classification systems for osteoarthritis of the ankle joint vary depending on the aetiology of the degenerative changes [17, 31]. Only 79 % of the optimally reduced ankle fractures showed good-to-excellent long-term outcome [30]. Post-traumatic osteoarthritis often seen after ankle fracture is dependent on several factors including fracture mechanism and joint instability [30, 33].

Classification systems exist to categorize radiographic signs of ankle osteoarthritis and could be useful to choose the most appropriate treatment and to predict the prognosis [15, 17, 30, 31]. The Kellgren classification has been used for the radiographic staging of osteoarthritis of the peritalar joints. Fair interobserver agreement was found [19]. Van Dijk et al. showed a high percentage of good-to-excellent results after arthroscopic removal of anterior impingement lesions in grade 0 and I osteoarthritis. The results were unsatisfactory in grade II osteoarthritis lesions [34]. Tanaka et al. found good long-term outcomes after low tibial osteotomy in varus osteoarthritis of the ankle in stages II and III. Patients with stage III or stage IV ankle osteoarthritis had persistent loss of joint space [31]. These studies suggested that treatment and prognosis are dependent on the stage of ankle osteoarthritis. Post-traumatic ankle arthritis can be a very disabling condition, and therefore, adequate treatment is helpful.

The purpose of this study is to identify the most reliable classification system for clinical outcome studies to categorize post-traumatic—fracture—osteoarthritis. We did assess the reliability of the classification systems coined by (1) van Dijk, (2) Kellgren, and (3) Takakura for post-traumatic—fracture—osteoarthritis in an online interobserver study. We hypothesized that the van Dijk osteoarthritis scale, the Kellgren classification, and the Takakura scale are not reliable.

## Materials and methods

The institutional research board (IRB) at the principal investigator’s hospital approved this study for the use of anonymized radiographs.

### Patient characteristics

Between 1974 and 2002, all patients with fractures that were treated with operative treatment in the Academic Medical Centre of Amsterdam, Level I trauma centre, were prospectively entered into a database according to the AO/OTA (Arbeitsgemeinschaft für Osteosynthesefragen/Orthopaedic Trauma Association) Comprehensive Classification of Fractures.

We identified a total of 437 AO/OTA-44 patients with fractures. Of these patients, 98 were deceased, 14 were classified as a cruris fracture, seven patients had an arthrodesis, five patients were considered mentally ill, three patients had a second fracture of the same ankle, one patient had an amputation of the affected leg, and one patient had a musculoskeletal disorder.

A total of 308 patients were eligible for long-term follow-up. Thirty-six patients were either emigrated or untraceable due to incorrect or outdated demographic data. The remaining 272 patients were invited for a long-term follow-up visit at our outpatient clinic. Of this group, 68 patients declined to participate in the study. Seventy-one patients did not respond and/or could not be contacted. A total of 133 patients participated in the study. The radiographs of five patients were not scored because the patients were not able to visit for follow-up radiographs. In total we included 128 patients in this study. Two patients had an AO/OTA type-A; one group A1, one group A2, zero group A3. Sixty-eight were AO/OTA type-B; zero group B1, zero group B2, 68 group B3 and there were 58 type 44-C fractures; nine group C1, 12 group C2, and 37 group C3.

The median follow-up time of the included 128 patients was 23 years (range 13–39 years).

There were a total of 128 patients, including 67 women and 61 men with a median age of 40 years (range 14–68 years) at time of the accident. The median age at follow-up was 63 years (range 36–92 years).

The median body mass index was 27 kg/m^2^, 17 (13 %) patients had diabetes, and 33 (30 %) smoked.

A fall [61 (48 %)] was the most common trauma mechanism, followed by fall from height [29 (22 %)], sports related injuries [22 (17 %)], traffic accident injuries [8 (6 %)], and fall of heavy weight on ankle [8 (6 %)]

Twenty-one (16 %) patients had a high-energy injury, five (4 %) fractures were open, and six (5 %) patients did have an ipsilateral leg injury.

We classified the fractures with the AO/OTA classification: 44A1: one (1 %), 44A2: one (1 %), 44A3: zero, 44B1: zero, 44B2: zero, 44B3: 68 (53 %), 44C1: nine (7 %), 44C2: 12 (9 %), 44C3: 37 (29 %).

A total of 108 patients had a bimalleolar ankle fracture, and 20 patients had a trimalleolar ankle fracture.

Seven ankle fractures were complicated with a post-operative infection, two patients had thrombosis, and one patient had non-union. Hardware removal took place in 93 (73 %) of the patients.

### Study design

Members of the Ankle Platform Study Collaborative Science of Variation Group were invited to evaluate 128 radiographs on a Web-based study platform (www.research.ankleplatform.com). All radiographs were weight bearing.

Each case had to be completed to continue with the next case. A total of 390 invitations were sent, and 150 members logged into our website. Observers were randomized to a custom Internet-based rating session with one of three set orders of cases (set order A, B, or C). A total of 118 observers completed the study (79 % of the initial responders).

After logon, the observers were asked general information about their practice. Observers then had to grade radiographic signs of ankle osteoarthritis according to the van Dijk osteoarthritis score [34], Takakura classification scale [17], and Kellgren classification [15] (ESM Appendix I).

### Variables, outcome measures, data sources, and bias

Independent variables were observer characteristics. Agreement among observers was determined using the multirater kappa measure described by Siegel and Castellan [27]. The multirater kappa measure is a frequently used statistical measure to describe chance-corrected agreement between ratings made by multiple observers (interobserver reliability) or between ratings made by one observer on multiple occasions (intra-observer reliability) [24]. The generated kappa values were interpreted according to the guidelines by Landis and Koch [18]: values of 0.010–0.20 indicate poor agreements; 0.21–0.40, fair agreement; 0.41–0.60, moderate agreement; 0.61–0.80, substantial agreement; and more than 0.81, almost perfect agreement. Zero indicates no agreement beyond that expected resulting from chance alone, −1.0 means total disagreement, and +1.0 represents perfect agreement.

Classifications were compared using the two-sample *Z*-test, and *p* values of <0.050 were considered significant [2, 4, 6, 7, 10–14, 20, 32, 36]. A *Z*-test was also used to compare subgroups. For a more intuitive understanding of presented data and to control for kappa paradox, the proportion of agreement, defined as the proportion of observers agreeing with the most provided answer, was calculated for each case.

The only incentive for observers to participate was group authorship.

This protocol was approved by the IRB of the Academic Medical Centre of Amsterdam, the Netherlands, under protocol number 2013_214#82013849.

### Statistical analysis

Post hoc power analysis showed a power of 80 % (*a* = 0.05; *B* = 0.20), with 90 observers reviewing 128 patients in three randomized groups of at least 30 observers with 42 cases, respectively [10].

## Results

### Participants

A total of 118 observers (93 % male, 7 % female) completed the online survey. A total of 37 observers participated in group A, 35 in group B, and 46 in group C. The majority of the observers who practised orthopaedics in continental Europe (53 %) were involved in resident training (78 %) and performed more than 30 ankle trauma surgeries a year (52 %) (Table 1). Only 6 % of the observers treated five or less ankle fractures per year.

**Table 1:** Observer demographics (*n* = 118)

Demographic	Number (%)
*Sex*	
Male	110 (93)
Female	8 (7)
*Area*	
Asia	16 (14)
Australia	1 (1)
Continental Europe	63 (53)
U K	9 (8)
Other	24 (20)
USA	5 (4)
*Years in practice*	
<5	31 (26)
5–10	37 (31)
11–20	39 (33)
21–30	8 (7)
>30	3 (3)
*Involvement in resident training*	
Yes	92 (78)
No	26 (22)
*Number of ankle cases treated per year*	
0–5	7 (6)
6–10	7 (6)
11–20	23 (19)
21–30	20 (17)
>30	61 (52)

### Interobserver agreement

The interobserver agreement between surgeons who participated in the survey was fair (reference value 0.21–0.40) for the van Dijk osteoarthritis scale (*k* = 0.24), and poor (reference value 0.01–0.20) for the Takakura classification (*k* = 0.18) and the Kellgren osteoarthritis scale (*k* = 0.19) (Table 2). In absolute means, 61 % agreement was achieved for the van Dijk osteoarthritis scale, 54 % agreement for the Takakura classification, and 50 % agreement for the Kellgren osteoarthritis scale.

**Table 2:** Interobserver agreement classifications

Classification	Categorical	*k* (*n* = 118)
Van Dijk	Fair agreement	0.24
Takakura	Poor agreement	0.19
Kellgren	Poor agreement	0.18

Comparison of the van Dijk osteoarthritis scale to the Takakura classification revealed a significant difference of kappa values (*p* < 0.001, CI 0.046–0.053), as well as comparison to the Kellgren osteoarthritis scale (*p* < 0.001, CI 0.056–0.064) with the high numbers of observers and cases included in this study.

### Factors associated with interobserver agreement

Poor-to-fair agreement was found for sex of the observers, years in practice, number of ankle fractures treated per year, involvement in resident training (Tables 3, 4, 5, 6). No particular factors increased the interobserver agreement. There was no significant difference between 5–10 years in practice and 11–20 years in practice for the van Dijk classification (*p* = 1.0). The interobserver agreement was not significantly higher with increasing numbers of ankle fractures treated per year for any of the classifications. There was a higher agreement in observers who are involved in resident training for all classifications (*p* < 0.001).

**Table 3:** Interobserver agreement for sex

Classification	Categorical	*k* 1 (*n* = 110)	Categorical	*k* 2 (*n* = 8)
Van Dijk	Fair agreement	0.24	Poor agreement	0.090
Takakura	Fair agreement	0.22	Poor agreement	0.17
Kellgren	Poor agreement	0.18	Poor agreement	0.10

1 = male; 2 = female

**Table 4:** Interobserver agreement for years in practice

Classification	Categorical	*k* 1	Categorical	*k* 2	Categorical	*k* 3	Categorical	*k* 4	Categorial	*k* 5
Van Dijk	Fair agreement	0.31	Fair agreement	0.23	Fair agreement	0.24	Fair agreement	0.21	Fair agreement	0.31
Takakura	Fair agreement	0.26	Fair agreement	0.21	Fair agreement	0.24	Poor agreement	0.10	Poor agreement	0.010
Kellgren	Poor agreement	0.18	Fair agreement	0.28	Fair agreement	0.21	Poor agreement	0.090	Fair agreement	0.22

1 = <5; 2 = 5-10; 3 = 11-20; 4 = 21-30; 5 = > 30

**Table 5:** Interobserver agreement for number of ankle fractures treated per year

Classification	Categorical	*k* 1	Categorical	*k* 2	Categorical	*k* 3	Categorical	*k* 4	Categorial	*k* 5
Van Dijk	Fair agreement	0.30	Fair agreement	0.25	Fair agreement	0.25	Poor agreement	0.20	Fair agreement	0.25
Takakura	Fair agreement	0.27	Fair agreement	0.24	Fair agreement	0.23	Poor agreement	0.14	Fair agreement	0.23
Kellgren	Poor agreement	0.15	Fair agreement	0.21	Fair agreement	0.17	Poor agreement	0.17	Poor agreement	0.18

1 = 0-5; 2 = 6-10; 3 = 11-20; 4 = 21-30; 5 = > 30

**Table 6:** Interobserver agreement for involvement in resident training

Classification	Categorical	*k* 1	Categorical	*k* 2
Van Dijk	Fair agreement	0.24	Poor agreement	0.20
Takakura	Fair agreement	0.24	Poor agreement	0.16
Kellgren	Poor agreement	0.19	Poor agreement	0.13

1 = yes; 2 = no

## Discussion

The most important finding of the present study was that the reliability of the van Dijk osteoarthritis scale, the Kellgren classification, and the Takakura scale was low.

Treatment and prognosis of post-traumatic—fracture—osteoarthritis of the ankle are suggested to be dependent on the stage of degenerative changes in the ankle joint [23, 31, 34, 35]. Therefore, reliable classification systems are important as they should guide treatment or prognosis to facilitate clinical decision-making and to compare patient cohort studies in the literature [28].

In this interobserver study, we did search for the most reliable classification system for post-traumatic—fracture—osteoarthritis. We did assess the reliability of the van Dijk osteoarthritis scale, the Kellgren classification, and the Takakura scale [15, 31, 34].

The strengths of this interobserver study include the large number of observers, which allowed randomization and subgroup analysis to increase the generalizability of the results. However, it should be interpreted in the light of several limitations. We have not studied a variety of potential sources of variation, including cultural differences, standardized training of observers, and computer and screen quality. This study was limited to interobserver agreement only because intra-observer agreement is less relevant to clinical practice as surgeons mostly agree with themselves and not so much with each other. In our survey, we did not find factors that increased interobserver agreement. We have not included patients that had an arthrodesis, and therefore, the patients in our cohort might have less severe arthrosis.

Interobserver agreement of the surgeons who participated in the survey was only fair for the van Dijk osteoarthritis scale (*k* = 0.24), and poor for the Takakura classification (*k* = 0.18) and Kellgren osteoarthritis scale (*k* = 0.19) (Table 2). Although the van Dijk osteoarthritis scale was significantly more reliable than the other classifications, the clinical relevance of this difference is debatable, because the interobserver agreement is low. Interestingly, more years of experience resulted in a higher interobserver agreement for all classifications; however, the number of treated ankle fractures per year did not influence interobserver agreement.

Since treatment and prognosis of post-traumatic—fracture—osteoarthritis of the ankle are suggested to be dependent on its stage [23, 31, 34, 35], reliable classification systems are helpful. In early stages, non-surgical treatment options such as anti-inflammatory medications or braces are often used [23, 35]. The main surgical treatment options include arthroscopic debridement and osteophyte resection, ankle osteotomy, total ankle prosthesis (TAP), and arthrodesis [3, 5, 8, 9, 21, 22, 25, 26, 29, 31, 34]. Arthroscopic debridement and osteophyte resection are recommended in mild osteoarthritis [8], and osteotomy is preferred in mild-to-moderate osteoarthritis with tibiotalar malalignment [29]. In end-stage osteoarthritis, TAP and arthrodesis are the treatment options of preference [3, 5, 21, 22, 26, 35].

Several classification systems should be compared to choose the most reliable classification system to prevent treatment variation. Krause et al. [16] tested the post-operative Canadian Orthopaedic Foot and Ankle Society end-stage ankle arthritis classification system in patients operated for end-stage ankle arthritis. This classification identifies no deformity, intra-articular deformity, extra-articular deformity, and surrounding joint arthritis. An almost perfect agreement was found (*k* = 0.89). A possible explanation for the higher interobserver agreement compared to our study could be the use of the Canadian Orthopaedic Foot and Ankle Society end-stage ankle arthritis classification system in end-stage ankle osteoarthritis patients only. Moreover, four observers evaluated 60 cases. The low number of observers can result in a higher kappa. Moreover, identifying osteoarthritis characteristics is much easier than identifying different stages of osteoarthritis that do not easily fall into categories.

Consistent with our study, the Kellgren and Lawrence osteoarthritis scale did show to be fair for classifying the degree of osteoarthritis present in the subtalar joint (*k* = 0.21) and talonavicular joint (*k* = 0.25) [19]. Treatment variation is unwanted in medical practice, and therefore, classification systems should have a high interobserver agreement to be reliable.

Van Dijk et al. described good-to-excellent results after arthroscopic removal of anterior impingement lesion low-grade osteoarthritis. The results were unsatisfactory in higher osteoarthritis lesions [34]. Tanaka et al. [31] found persistent loss of joint space in higher-stage ankle osteoarthritis compared to early stage ankle osteoarthritis.

## Conclusions

This study documents fair interobserver agreement for the van Dijk osteoarthritis scale, and poor interobserver agreement for the Takakura and the Kellgren osteoarthritis classification systems. Because of the low reliability of all three investigated classification systems in this study and a substantial percentage of surgeons who disagreed, those classifications cannot be used in day-to-day practice in terms of clinical decision-making. Easier classifications with fewer categories might result in higher reliability and are therefore more valuable for clinical practice.

## Electronic supplementary material

Below is the link to the electronic supplementary material.

Supplementary material 1 (XLSX 49 kb)
